# Attempted Suicide in the Older Adults: A Case Series From the Psychiatry Ward of the University Hospital Maggiore Della Carità, Novara, Italy

**DOI:** 10.3389/fpubh.2021.732284

**Published:** 2022-01-03

**Authors:** Carla Gramaglia, Maria Martelli, Lorenza Scotti, Lucia Bestagini, Eleonora Gambaro, Marco Romero, Patrizia Zeppegno

**Affiliations:** ^1^Institute of Psychiatry, Department of Translational Medicine, University of Piemonte Orientale, Novara, Italy; ^2^S.C. Psichiatria, Azienda Ospedaliero Universitaria Maggiore della Carità, Novara, Italy; ^3^Department of Translational Medicine, University of Piemonte Orientale, Novara, Italy

**Keywords:** suicide attempt, elderly, Psychiatry Ward, gender, multiple attempters

## Abstract

**Introduction:** As suicide rates increase with age, it is mandatory to carefully assess old age suicidal behaviors. Our aim was to describe the main socio-demographic and clinical features of a sample of suicide attempters aged 65 years and older, and to assess differences within the sample (men vs. women; patients with vs. without a previous history of suicide attempt; patients with vs. without a previous psychiatric history).

**Methods:** Retrospective study conducted at the Maggiore della Carità University Hospital, Novara, Italy.

**Results:** A higher percentage of female patients in our sample were treated by or referred to mental health services, while a greater percentage of male patients required a prolonged clinical observation in the Emergency Room (ER) or in non-psychiatric wards before psychiatric admission. The percentage of patients without previous psychiatric history taking anxiolytic and sedative medications was 25%.

**Conclusion:** It is likely that different clusters and types of suicide attempters exist. Women in our sample appeared more proactive in asking for help, and more likely to be already treated by or referred to a psychiatric service, suggesting the need to facilitate the access to psychiatric services for the male population aged 65 years and older, or to offer support and care for the non-psychiatric reasons (comorbidities, pain, and loss of autonomy) possibly underlying suicidal behavior in this specific group. The use of medications deserves more attention considering the possible critical diagnostic issues in this age group.

## Introduction

Suicide is a worldwide phenomenon that accounts for almost 800,000 deaths per year (1–6). The number of non-lethal suicide attempts is much greater, with estimates suggesting that there are close to 20 suicide attempts for every completed suicide (3). A suicide attempt can be defined as an “engagement in potentially self-injurious behavior, in which there is at least some intent to die” (4); it is an individual act performed with the intention to end one's life, but which does not result in death (7). On the other hand, suicide ideation (SI) is reported by 2% of people every year (3) and can be defined as a spectrum of thoughts spanning from thoughts and wishes about wanting to end life with no intent, to suicide plans (8, 9).

Two peaks of overall suicidal behaviors can be identified: the first in young adults and the second in the older ones (6, 10, 11); nonetheless, as older adults more frequently die by suicide compared with younger adults, they account for a disproportionately high number of suicide deaths (12). This is likely related to the evidence that suicide attempts among individuals aged 65 years and older are characterized by lower impulsivity and higher lethal intent (6, 10). As far as gender is concerned, it is widely acknowledged that women are at higher risk of self-injurious thoughts (3, 10), whereas men have higher suicide rates (2, 3, 5, 10, 13, 14). This gender difference largely depends on the choice of suicide method (men tend to use more violent and lethal ones, such as firearms and hanging, while women use drowning or self-poisoning) and reaches a peak among older adults (3, 5), where the male/female suicide death ratio is 6.63 for those aged 75–79 years and reaches up to 12.80 for people over the age of 85 years (3).

Among the psychosocial risk factors for suicidal behaviors in this age group, there may be weak social networks, living alone, a condition of social isolation, lack of routine and social activities, and low socio-economic status; all these factors may eventually lead to hopelessness and helplessness, or to the loss of meaning in life (6–8, 10, 11). Furthermore, bereavement and becoming a widow/widower should be considered as independent risk factors (7, 10, 11).

Suicidality in older adults has been linked to some features of this specific period of life, which are a consequence of aging, such as physical illness and comorbidities (e.g., cancer, liver disease, neurologic disorder, such as dementias, genital disorders, or rheumatoid disorders) (2, 8, 11, 15), functional disabilities (6), and pain. The relationship with suicidality is especially strong when comorbidities and pain significantly impact on the individual's independence, autonomy, and dignity, and consequently decrease quality of life, but also pleasure, sense of meaning and purpose in life, perceived personal value and self-esteem (16–18). Actually, approximately 55% of late life suicides are associated with physical illness (19, 20) and a relationship between suicidal risk in older adults and pain intensity has been suggested (15).

Moreover, in older age there is a high risk of unrecognized and untreated psychiatric illnesses (21, 22) but even though depression can be associated with late life suicide (19, 23–25), most depressed older adults do not become suicidal. A study carried out on a population of 97-years old subjects found that most of those experiencing suicidal feelings (77%) did not match criteria for depression. On the other hand, the possibility of misdiagnosing or underdiagnosing depressive symptoms in older adults should not be overlooked (10, 11, 13, 14). Anxiety disorders, in association with major depressive disorder (MDD), represent another important psychiatric comorbidity, being found in 16.6% of suicides in this age group (13).

In addition, clinicians dealing with suicidal behavior in older adults should be aware of the debated concept of rational suicide (6, 26–28) and of ageism-related prejudices, which could lead to underestimate depression and suicidal risk in this age group (6, 29, 30). From a clinical standpoint, an ageist attitude may lead clinicians to consider depression understandable in the context of health and living circumstances of an older person, and suicides of an older adults as rational choices (26).

As suicide rates increase with age, and considering worldwide aging trends, it is mandatory to carefully assess the phenomenon of old age suicidal behaviors (19, 20, 27).

As stated above, older adults more frequently die by suicide compared with younger adults, and their suicide attempts are characterized by lower impulsivity and higher lethal intent. As suicide attempts are the most important predictor for a later suicide (28, 29), we focused on suicide attempts of older adults in the Psychiatry Ward, and we compared patients with and without a previous suicide attempt for the same reason. Furthermore, as there are widely acknowledged gender differences in suicidal behaviors (2, 3, 5, 10, 13, 14, 30), we compared male and female suicide attempters. Last, as suicidal behaviors are reported as more frequent in individual with a psychiatric diagnosis (31), we compared patients with and without a psychiatric history.

Summarizing, our aim with this study was to describe the main socio-demographic and clinical features of a sample of suicide attempters aged 65 years and older; furthermore, according to the above-mentioned data from the literature, to assess the possible differences between men and women; patients with and without a previous history of suicide attempt; and patients with and without a previous psychiatric history.

## Methods

We performed a cross-sectional study based on medical charts of patients admitted to the Psychiatric Ward of the “Maggiore della Carità” University Hospital in Novara, Italy, specifically focusing on admissions for attempted suicide in the population of patients aged 65 years or older.

The “Maggiore della Carità” University Hospital is the main referral hospital for all North-Eastern Piedmont and the second for dimension in Piedmont; its catchment area can be considered representative for the whole region. It has a high specialization level Emergency Room (ER), that admits about 60,000 adult people per year. Patients are assessed with a triage procedure, and a priority code is attributed by the nurse. According to this code, the patients are then evaluated by an emergency physician that asks for psychiatric consultation after a preliminary assessment, according to the hospital guidelines for the ER and considering clinical conditions of patients (30). As the hospitalization of a patient residing out of this catchment area is a sporadic event that occurs only when there are no available beds in another psychiatric ward, our sample included only patients from north-eastern Piedmont, who anyway are not expected to be different from those accessing other ERs in other Hospitals in Piedmont.

Our Psychiatry Ward is in the General University Hospital, and it admits acutely ill psychiatric patients affected by all type of psychiatric diseases, usually after assessment in the ER. As described above, patients in the ER are evaluated by ER clinicians from a physical standpoint (with the aid of general exams, such as blood count, urine drugs test, electrocardiogram, and all other exams required according to clinical judgement), and receive the psychiatric consultation. In case of attempted suicide that requires an immediate admission to intensive care unit (ICU), the psychiatric evaluation is not performed in the ER, but later, when the patient is stabilized and can be eventually admitted to the Psychiatry Ward.

The inclusion criteria for this study were the following: (1) hospitalization in the Psychiatric Ward in the period 2016–2020 for an attempted suicide; and (2) age of 65 years or older. As described in the introduction, we considered attempted suicide as any self-injurious behavior with at least some intent to die (4). No specific exclusion criteria were adopted. For this research project, we gathered information from the following sources: discharge summaries, medical records and medical charts, the hospital applications for the consultation of ER records (PSnet), and medication prescription (Bustermed) during hospital admission and at discharge. Medical records between 2018 and 2020 were available as online documents (CSA website), while for previous records we consulted the paper documents stored in a warehouse in Biandrate (Novara, Italy).

**Figure 1:**
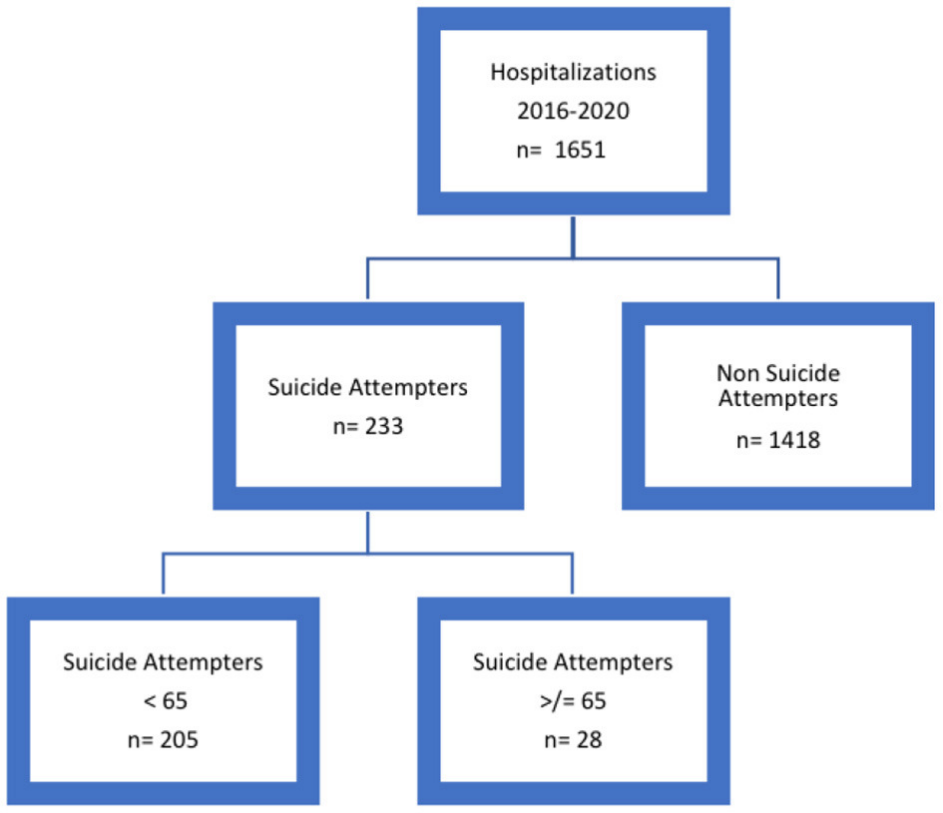
Selection process of records and discharge summaries of patients.

First, we retrospectively and carefully analyzed all the discharge summaries collected in the Psychiatry Ward from January 1, 2016 to December 31, 2020, to allow the identification of all the admissions for attempted suicide. Then, we grouped all the hospitalizations in two categories: “suicide attempters” and “non-suicide attempters.” Afterwards, we screened all the records concerning the “suicide attempters” category to identify patients aged 65 years or older, creating two subgroups: “suicide attempters aged less than 65 years” and “suicide attempters aged more than 65 years.” At the end of this selection process, we eventually identified 28 patients, aged 65 years or older, who were admitted for a suicide attempt to the Psychiatry Ward during the study period (as shown in Figure 1).

From the medical charts and records, we gathered the available information about history of patients, ER access, physical conditions at presentation, data about hospitalization, and discharge. With more detail, we gathered data about the following variables: socio-demographic (date of birth, age, gender, with whom the patient lives, educational level, and marital status), clinical history (previous psychiatric diagnosis, being followed by psychiatric services, psychiatric therapy, organic comorbidities, previous psychiatric hospitalizations, previous suicide attempts, history of alcohol or substance abuse, family history of psychiatric diseases, and comorbidity with dementia), ER access (ER access mode, who called for help, Glasgow Coma Scale at ER admission, suicide method, need for emergency medical therapy, need for hospitalization in another clinical ward different from the Psychiatry Ward or more than 12 h observation in the ER, and outcome of urine drug test), hospitalization and discharge [date of hospitalization and discharge, duration of inpatient treatment, mandatory/compulsory medical treatment, Nurses Global Assessment of Suicide Risk (NGASR) (32) score at admission, participation to group rehabilitation activities, need for physical revaluations as a consequence of the suicide attempt, diagnosis and medication prescription at discharge, and further referral to a psychiatric service after hospital discharge].

We checked for possible multiple hospitalizations of the same patients, but then every personal information (name and surname) was removed, patients were assigned a numerical code and all the information we gathered was anonymously recorded in an Excel data sheet.

As this research did not entail any procedure beyond everyday clinical practice, the need for approval on behalf of the Institutional Review Board was waived.

### Data Analysis

Descriptive statistics were used to summarize the characteristics collected on the study's subjects. Categorical variables were reported as absolute frequencies and percentages and continuous variables as median and first and third quartiles. The Fisher's exact test was used to assess the differences between characteristics of patients in the following subgroups: men and women; patients with and without a previous history of suicide attempt; and patients with or without a previous psychiatric history. For continuous variables, the differences between groups were assessed using the Mann–Whitney test. The evaluation of the differences between groups were performed when at least 5 subjects for each level of the categorical variables were available except for specific variables of interest.

Data were analyzed using SAS version 9.4 (SAS Institute Inc.Cary, NC, USA) (33).

## Results

### Description of the Sample

In this retrospective study, we analyzed a total of 1,651 discharge letters according to the criteria described in the methods, and we eventually included a sample of 28 suicide attempters aged 65 years and older (as shown in the flowchart in Figure 1 for the selection procedure).

At discharge, diagnoses were the following: acute stress reaction (11, 39.3%), mood disorder (11, 39.3%), and cognitive decline with or without depression diagnosis (6, 21.4%). More specifically, among patients with a discharge diagnosis of mood disorder, 4 had an adjustment disorder with depressed mood, 3 had a diagnosis of MDD, single episode, 2 had a diagnosis of MDD, single episode, severe, specified as with psychotic behavior, and 2 had an unspecified episodic mood disorder.

Table 1 reports the distribution of the socio-demographic and clinical characteristics of the patients included in the study. The sample was composed by 17 women (60.7%) and 11 men (39.3%), with a median age of 72.5 years (Q1–Q3:71.0–79.5). Most patients lived with their family (19, 67.9%), 8 lived alone (28.6%), and one (3.6%) in a residential facility. Eighteen patients were married (64.3%), 8 (28.6%) were widowed; information about marital status was not available for 2 patients (7.1%). As far as educational level is concerned, 19 patients (67.9%) had a secondary school graduation, while 2 (7.1%) had a high school or university degree, one (3.6%) patient was illiterate; and for 6 patients (21.4%), this information was missing. Family history of psychiatric diseases was negative for 12 patients, while 9 of them (32.1%) reported the presence of a psychiatric disorder in their family; and this information was not available for the other 7 patients (25.0%).

**Table 1:** Sociodemographic, hospitalization, and discharge features for the whole sample (*N* = 28).

	**Median**	**(Q1-Q3)**
**Age**	**72.5**	**(71.0–79.5)**
	**N**	**%**
**Sex**		
*Males*	11	39.29
*Females*	17	60.71
**Housing**		
*Alone*	8	28.57
*Family*	19	67.86
*Care facilities*	1	3.57
**Marital status**		
*Married*	18	69.23
*Widow*	8	30.77
*Missing*		2
**Education level**		
*Illiterate*	1	4.55
*Primary/Middle School*	19	86.36
*High School/University*	2	9.09
*Missing*		6
**Family history of psychiatric disease**		
*No*	12	57.14
*Yes*	9	42.86
*Missing*	7	
**Previous Psychiatric history**		
*No*	8	28.57
*Yes*	20	71.43
**Diagnosis**		
*Anxious depressive syndrome/depression*	14	87.5
*Other*	2	12.5
*Missing*		4
**Treated by a mental health service at the time of ER access**		
*No*	13	46.43
*Yes*	15	53.57
**Type of service**		
*Community mental health service*	12	80
*Others health services*	3	20
**Psychopharmacological medication at the time of ER access**		
*No*	10	35.71
*Yes*	18	64.29
**Type of medication**		
*Only antidepressant*	5	27.78
*Only anxiolitics or sedatives*	7	38.89
*Antidepressant and sedatives*	6	33.33
	***N** **=*** **28**	
	**Median**	**(Q1-Q3)**
**Previous Psychiatric hospitalization**		
*No*	18	64.29
*Yes*	10	35.71
**Number of previous hospitalizations**		
*1*	5	50
*>1*	5	50
**History of suicide attempts**		
*No*	19	67.86
*Yes*	9	32.14
**Number of suicide attempts**		
*1*	7	77.78
*>1*	2	22.22
**Comorbidities**		
*No*	3	10.71
*Yes*	25	89.29
**Number of comorbidities**		
1	8	32.00
>1	17	68.00
**Type of ER access**		
*Autonomous*	16	72.73
*Ambulance*	6	27.27
*Missing*	6	
**Helps called by**		
*Family members/neighbors*	8	28.57
*Medical staff*	3	10.71
*Missing*		17
**Suicide attempt methods**		
*Medications abuse*	12	42.86
*Poisoning*	1	3.57
*Edged weapon*	6	21.43
*Falling from height*	4	14.29
*Hanging*	1	3.57
*Other*	4	14.29
**Urgent therapy for suicide attempt**		
*No*	10	35.71
*Yes*	18	64.29
**Substances use**		
*No*	1	5.88
*Yes*	16	94.12
*Missing*	11	
	***N** **=*** **28**	
**Hospital admission year**		
*2016*	4	14.81
*2017*	5	18.52
*2018*	4	14.81
*2019*	5	18.52
*2020*	*9*	33.33
*Missing*		1
**Need for more than 12 hours observation in clinical**		
**non-psychiatric ward**		
No	17	60.71
Yes	11	39.29
**Need of somatic revaluation during hospitalization**		
*No*	17	60.71
*Yes*	11	39.29
**Congnitive impaiment**		
*No*	21	75
*Yes*	7	25
**Medication prescription at discharge**		
*No*	1	3.57
*Yes*	27	96.43
**Type of medication**		
*Only antidepressant*	3	10.71
*Only anxiolitics or sedatives*	13	46.43
*Combination of antidepressant and anxiolytics*	11	39.29
**Referral to a psychiatric service at discharge**		
*No*	2	7.14
*Yes*	26	92.86
**Service**		
*Mental health service*	11	39.29
*Other psychiatric ward*	3	10.71
*Nursing home*	10	35.71
*Other*	2	7.14

Most patients had a previous diagnosis of a psychiatric disorder (20, 71.4%), that was an anxious or depressive disorder in most cases (14, 50%) and “other diagnosis” in 2 cases (7.1%; personality disorder in one case and dementia in the other). Fifteen patients (53.6%) were currently treated by a mental health service at the time of ER access; with more detail, 12 patients (42.9%) were followed by the community mental health service and 3 (10.7%) by other mental health services, such as private practice psychiatrist or psychologist or addiction services. Eighteen (64.3%) patients were taking a psychopharmacological therapy when they attempted suicide: anxiolytic drugs only, antidepressants only, and both these medications in 7 (25%), 5 (17.9%), and 6 cases (21.4%), respectively. Most patients were never admitted to a Psychiatry Ward before (18, 64.3%); 9 patients (32.1%) had a previous history of attempted suicide, while 2 patients (7.1 %) had more than one lifetime suicide attempt. Regarding somatic disorders, most patients had an organic comorbidity (25, 89.0%), and 17 (60.7%) of them suffered of more than one organic disease.

Emergency Room access was with an ambulance for most patients (16, 57.1%), though it was not usually possible to go back to who called the emergency number (17, 60.7%); this information was available in 8 (28.6%) cases, where it was relatives or friends who called the rescue number. Police intervention was necessary in minority of cases (3, 10.7%).

The most frequent suicide attempt method was drug ingestion (12, 42.9%), followed by edged weapons (6, 21.4%), precipitation (4, 14.3%), and other methods (4, 14.3%; one drowning, one attempted drowning, one strangulation, one stopped eating); last, one patient attempted suicide by hanging (3.6%) and one by poisoning (3.6%).

For 16 (57.1%) patients, it has contacted the Poison Control Center in Pavia, Italy, which is a center specialized in consulting on emergency therapy targeted to the specific poison taken by the patient. An urgent therapy in the ER was required in 18 cases (64.3%), and in most cases it was a gastric lavage (8, 29.6%). Eleven (39.3%) patients needed to spend more than 12 h in a non-psychiatric ward to complete the observation required for somatic reasons. Drug urine test was negative in most cases (16, 57.1%); in 11 (39.3%) cases, this information was not available; last, only one resulted positive (barbiturates).

In the study period, the number of admissions per year was not homogeneously distributed; there were 4 (14.3%) hospitalizations in 2016, 5 (17.9%) in 2017, 4 (14.3%) in 2018, 5 (17.9%) in 2019, and 10 (35.7%) in 2020. Most patients accessed the ER in spring (10, 35.7%), 8 in winter (28.6%), 7 in summer (25%), and 3 in autumn (10.7%).

Table 2 reports the distribution of characteristics of patients according to gender, previous history of suicide attempt and previous psychiatric history, the *p* of the test used for the group comparison, the prevalence ratio, and corresponding 95% *CI*.

**Table 2:** Results from comparison by gender, previous history of suicide attempt and previous psychiatric history.

	**Sex**				**Previous suicide attempts**				**Previous psychiatric history**			
	**Males *N* = 11**	**Females *N* = 17**	**Fisher exact test *p*-value**	**PR (95%CI)**	**No *N =* 19**	**Yes *N =* 9**	**Fisher exact test *p*-value**	**PR (95%CI)**	**No *N =* 8**	**Yes *N =* 20**	**Fisher exact test *p*-value**	**PR (95%CI)**
	**N (%)**	**N (%)**			**N (%)**	**N (%)**			**N (%)**	**N (%)**		
**Age, median (Q1-Q3)**	77 (72–80)	72 (69–76)	0.1548^*^	0.97 (0.92–1.03)	76 (72–80)	69 (68–71)	0.0063^*^	0.83 (0.71–0.97)	79.5 (75.5–87.5)	72 (69–76)	0.0086^*^	0.94 (0.90–0.98)
**Sex**												
Males					10 (52.63)	1 (11.11)	0.0491	1	3 (37.5)	8 (40)	1.0000	1
Females					9 (47.37)	8 (88.89)		5.18 (0.76–35.86)	5 (62.5)	12 (60)		0.97 (0.60–1.56)
**Marital status**												
Married	7 (63.64)	11 (64.71)	0.6828	1	12 (63.16)	6 (66.67)	1.0000	1	4 (50)	14 (70)	0.5240	1
Widow	4 (36.36)	4 (23.53)		0.82 (0.37–1.79)	6 (31.58)	2 (22.22)		0.92 (0.30–2.81)	3 (37.5)	5 (25)		0.83 (0.46–1.50)
**Family history of psychiatric disease**												
No	3 (27.27)	9 (52.94)	1.0000	1	8 (42.11)	4 (44.44)	0.0809	1	3 (37.5)	9 (45)	0.2016	1
Yes	3 (27.27)	6 (35.29)		0.89 (0.51–1.57)	4 (21.05)	5 (55.56)		1.43 (0.62–3.30)	1 (12.5)	8 (40)		0.86 (0.63–1.16)
**Alcohol consumption in the past**												
*No*	7 (63.64)	12 (70.59)	1.0000	1	14 (73.68)	5 (55.56)	0.4074	1	7 (87.5)	12 (60)	0.2144	1
*Yes*	4 (36.36)	5 (29.41)		0.88 (0.45–1.73)	5 (26.32)	4 (44.44)		1.69 (0.59–4.82)	1 (12.5)	8 (40)		1.41 (0.93–2.13)
**Previous Psychiatric history**												
*No*	3 (27.27)	5 (29.41)	1.0000	1	8 (42.11)	0 (0.00)	0.0292	NA				
*Yes*	8 (72.73)	12 (70.59)		0.96 (0.50–1.83)	11 (57.89)	9 (100)						
**Treated by a mental health service at the time of ER access**												
*No*	6 (54.55)	7 (41.18)	0.7000	1	10 (52.63)	3 (33.33)	0.4348	1	8 (100)	5 (25)	0.0004	1
*Yes*	5 (45.45)	10 (58.82)		1.24 (0.67–2.30)	9 (47.37)	6 (66.67)		1.73 (0.54–5.59)	0 (0.00)	15 (75)		2.60 (1.31–5.17)
**Comorbidities**												
*No*	0 (0.00)	3 (17.65)	0.2579	1	1 (5.26)	2 (22.22)	0.2344	1	1 (12.5)	2 (10)	1.0000	1
*Yes*	11 (100.00)	14 (82.35)		0.56 (0.40–0.80)	18 (94.74)	7 (77.78)		0.42 (0.15–1.16)	7 (87.5)	18 (90)		1.08 (0.47–2.49)
**Currently taking Psychopharmacological medication at the time of ER access**												
*No*	6 (54.55)	4 (23.53)	0.1245	1	9 (47.37)	1 (11.11)	0.0980	1	6 (75)	4 (20)	0.0110	1
*Yes*	5 (45.45)	13 (76.47)		1.81 (0.80–4.06)	10 (52.63)	8 (88.89)		4.44 (0.65–30.62)	2 (25)	16 (80)		2.22 (1.02–4.83)
**Previous Psychiatric hospitalization**												
*No*	9 (81.82)	9 (52.94)	0.2264	1	17 (89.47)	1 (11.11)	0.0001	1	8 (100)	10 (50)	0.0251	1
*Yes*	2 (18.18)	8 (47.06)		1.60 (0.92–2.79)	2 (10.53)	8 (88.89)		14.4 (2.09–99.19)	0 (0.00)	10 (50)		1.80 (1.19–2.72)
**History of suicide attempts**												
*No*	10 (90.91)	9 (52.94)	0.0491	1					8 (100)	11 (55)	0.0292	1
*Yes*	1 (9.09)	8 (47.06)		1.88 (1.11–3.18)					0 (0.00)	9 (45)		1.73 (1.18–2.54)
**Type of ER access**												
*Autonomous*	1 (12.5)	5 (35.71)	0.3512	1	3 (17.65)	3 (60)	0.1005	1	1 (12.5)	5 (35.71)	0.3512	1
*Ambulance*	7 (87.5)	9 (64.29)		0.68 (0.39–1.18)	14 (82.35)	2 (40)		0.25 (0.05–1.15)	7 (87.5)	9 (64.29)		0.68 (0.39–1.18)
**Emergency therapy for suicide attempt**												
*No*	2 (18.18)	8 (47.06)	0.2264	1	5 (26.32)	5 (55.56)	0.2096		1 (12.5)	9 (45)	0.1937	1
*Yes*	9 (81.82)	9 (52.94)		0.63 (0.36–1.09)	14 (73.68)	4 (44.44)		0.44 (0.15–1.29)	7 (87.5)	11 (55)		0.68(0.44–1.04)
**Need for more than 12 hours observation in clinical non-psychiatric ward**												
No	4 (36.36)	13 (76.47)	0.0527	1	10 (52.63)	7 (77.78)	0.2495	1	4 (50)	13 (65)	0.6715	1
Yes	7 (63.64)	4 (23.53)		0.48 (0.21–1.09)	9 (47.37)	2 (22.22)		0.44 (0.11–1.75)	4 (50)	7 (35)		0.83 (0.50–1.40)
**Duration of the hospitalization**												
≤ *2 weeks*	7 (63.64)	9 (52.94)	0.7047	1	10 (52.63)	6 (66.67)	0.6870	1	5 (62.5)	11 (55)	1.0000	1
*>2 weeks*	4 (36.36)	8 (47.06)		1.19 (0.66–2.14)	9 (47.37)	3 (33.33)		0.67 (0.21–2.14)	3 (37.5)	9 (45)		1.09 (0.69–1.74)
**Need of somatic revaluation during hospitalization**												
*No*	3 (27.27)	14 (82.35)	0.0062	1	9 (47.37)	8 (88.89)	0.0491	1	4 (50)	13 (65)	0.6715	1
*Yes*	8 (72.73)	3 (17.65)		0.33 (0.12–0.89)	10 (52.63)	1 (11.11)		0.19 (0.03–1.34)	4 (50)	7 (35)		0.83 (0.50–1.40)
**Congnitive impaiment**												
*No*	7 (63.64)	14 (82.35)	0.3809	1	12 (63.16)	9 (100)	0.0621	NA	5 (62.5)	16 (80)	0.3715	1
*Yes*	4 (36.36)	3 (17.65)		0.64 (0.26–1.59)	7 (36.84)	0 (0.00)			3 (37.5)	4 (20)		0.75 (0.38–1.49)
**Type of medication at discharge**												
*Only antidepressant*	2 (20.00)	1 (5.88)	0.0249	0.72 (0.13–3.97)	2 (11.11)	1 (11.11)	0.6224	1.44 (0.22–9.50)	1 (14.29)	2 (10)	1.0000	0.87 (0.37–2.04)
*Only anxiolitics or sedatives*	7 (70.00)	6 (35.29)		1	10 (55.56)	3 (33.33)		1	3 (42.86)	10 (50)		1
*Combination of antidepressant* *and anxiolytics*	1 (10.00)	10 (58.82)		1.97 (1.06–3.65)	6 (33.33)	5 (55.56)		1.97 (0.60–6.44)	3 (42.86)	8 (40)		0.95(0.59–1.51)
**Referral to a psychiatric service at discharge**												
*No*	1 (9.09)	1 (5.88)	0.0138	0.58 (0.14–2.37)	1 (5.26)	1 (11.11)	0.0019	NA	0 (0.00)	2 (10)	0.1533	1.167(0.94–1.45)
*Mental health service/* *Another psychiatric ward*	2 (18.18)	12 (70.59)		1	6 (31.58)	8 (88.89)			2 (25)	12 (60)		1
*Nursing home/Other*	8 (72.73)	4 (23.53)		0.39 (0.17–0.89)	12 (63.16)	0 (0.00)			6 (75)	6 (30)		0.58 (0.32–1.07)
**Diagnosis at discharge**												
*Acute reaction to stress*	4 (36.36)	7 (41.18)	0.7870	1	7 (36.84)	4 (44.44)	0.7635	1	3 (37.5)	8 (40)	0.4832	1
*Mood disturbance*	4 (36.36)	7 (41.18)		1.00 (0.53–1.88)	7 (36.84)	4 (44.44)		1.00 (0.33–3.02)	2 (25)	9 (45)		1.13 (0.71–1.78)
*Cognitive decline with or* *without depression*	3 (27.27)	3 (17.65)		0.79 (0.31–1.97)	5 (26.32)	1 (11.11)		0.46 (0.07–3.23)	3 (37.5)	3 (15)		0.69 (0.29–1.66)
**NSGAR score**												
*High*	4 (44.44)	2 (14.29)	0.2239	0.46 (0.14–1.47)	3 (18.75)	3 (42.86)	0.3797	NA	0 (0.00)	6 (37.5)	0.0642	NA
*Mid*	4 (44.44)	11 (78.57)		1	11 (68.75)	4 (57.14)			7 (100)	8 (50.00)		
*Low*	1 (11.11)	1 (7.14)		0.68 (0.17–2.82)	2 (12.5)	0 (0.00)			0 (0.00)	2 (12.50)		

* *Mann–Whitney test*.

Subjects with a history of suicide attempts and those treated with a combination of antidepressant and anxiolytics at discharge were more likely to be women compared with those without history of suicide attempts or treated with anxiolytics or sedatives only (*p* 0.0491 and 0.0249, respectively). Conversely, subjects needing somatic revaluation during hospitalization and referring to nursing home or other services at discharge were less likely to be women compared with subjects without somatic revaluation or those referring to mental health service or other psychiatric wards (*p* 0.0062 and 0.0138, respectively).

Previous suicide attempts were more likely to occur in patients with previous psychiatric hospitalization (*p* 0.0001) or with previous psychiatric history (*p* 0.0292) compared with those without previous hospitalizations and no psychiatric history; moreover, they were more likely to be referred to a mental health service or to another Psychiatric Ward at discharge (*p* 0.0019), compared with other services.

Finally, previous psychiatric history was more common among subjects treated by a mental health service at the time of ER access (*p* 0.0004), taking psychopharmacological medication at the time of ER access (*p* 0.0110), having previous psychiatric hospitalization (PR 1.80), and having a history of suicide attempts (*p* 0.0251) compared with those who did not have these features. Regarding age, patients with previous psychiatric history and suicide attempts seemed younger than those without these characteristics (*p* 0.0086 and 0.0063, respectively).

## Discussion

Old age suicide is a complex phenomenon deserving attention both from a research and clinical standpoint, considering the worldwide aging trends. In the 5-years period we assessed, there was a total of 223 admissions for suicide attempt, and 12% of these suicide attempts involved patients aged 65 years and older. According to the literature, it is known that suicide is more common in older adults than in younger ones—likely due to lower impulsivity and higher lethal intent among older adults, while suicide attempts are more frequent in younger adults (10, 12, 34). This might explain the small percentage of older adults with a suicide attempt admitted to our Psychiatry Ward. The median age of patients with a previous suicide attempt was lower than that of patients with no history of previous suicide attempts. Nonetheless, we could not retrieve data about all the suicide attempters who were not admitted to the Psychiatry Ward, hence our results need to be considered in the light of the intrinsic limitations of the study design.

### Comparison Between Male and Female Suicide Attempters Admitted to the Psychiatry Ward During the Study Period

In our sample, most suicide attempters were women. Actually, in the literature an important gender difference has been described regarding suicidal behavior, known as the “gender paradox of suicidal behavior” (35). This refers to an over-representation of women in non-fatal suicidal behavior and a preponderance of men in completed suicide: not only suicidal methods are different between men and women, but there are gender differences in the likelihood of reporting suicide and in help seeking. Further gender differences have been suggested, with men perceiving more self-stigma and women perceiving more shame (36).

At discharge, compared with men, a higher percentage of female patients was prescribed a combined treatment with both antidepressants and anxiolytic medications, rather than anxiolytics/sedatives alone, and suggested referral to mental health services. The results we found can depend on the fact that, compared with men, women in our sample seemed to be more likely to have a problem deserving a psychiatric treatment and follow-up (this may also be the reason why more women than men were referred to community mental health centers at discharge). Women may be more likely than men to use the healthcare system resources when experiencing psychological distress (37, 38). Regarding medications, a paper describing the patterns of psychotropic use immediately before and after ER presentation found that about half of subjects presenting to the ER for suicidal behavior, suicidal ideation, or non-suicidal self-injury, had received an antidepressant prescription before the ER access, with a spike in new prescriptions in the previous month (39).

In our sample, male patients more frequently required more revaluations of their somatic condition during their hospital stay, compared with female patients. The interpretation of this finding can be 2-fold. On the one hand, it may suggest a greater suicide intent on behalf of male patients, that leads them to more severe suicide attempts and to the choice of more violent or high-lethality methods: from this standpoint, the need for revaluations may be a consequence of the severity of the suicide attempt. Data in the literature are consistent with the fact that women engage in non-fatal suicidal behaviors more frequently than men (2, 3, 10); indeed, the latter tend to adopt more violent methods, such as hanging and firearms, while women more frequently attempt suicide with self-poisoning (3). On the other hand, the need of revaluations of the somatic conditions may depend on a greater fragility of the baseline conditions of the suicide attempters, which could be responsible of the more frequent discharge to nursing facilities, where patients can receive adequate treatment for their medical conditions. Nonetheless, we failed to find differences in organic comorbidities between the two groups, but we should underscore that we just accounted for presence/absence of comorbidities and for number of comorbidities, while we were not able to classify them according to severity. Furthermore, it is not only the severity of the organic comorbidity which impacts on the quality of life of patients, but how the illness is experienced and coped with, and regrettably we have no possibility to discuss about these issues as these were not assessed with an objective or measurable approach. Anyway, if this was the case (worst somatic conditions in male suicide attempters, notwithstanding the severity of the suicide attempt), it could be hypothesized that while in women, the suicide attempt may be a way to ask for help on behalf of a person with psychological distress, in men, the suicide attempt may be related to worse somatic conditions and to the difficulties in coping with them. Nonetheless, it cannot be excluded an underestimation of depression in men, which may derive from an atypical presentation of the disorder and from the peculiar help-seeking behavior of men; both the attitude of male patients and beliefs of mental health professionals about them may be influenced by normative expectations regarding the male gender (40).

### Comparison Between Patients With and Without a Previous Suicide Attempt Admitted to the Psychiatry Ward During the Study Period

Apart from expected differences in previous psychiatric history, admissions to a Psychiatry Ward and further referral to mental health services at discharge, our results suggest that patients with no previous history of suicide attempt required more somatic revaluations and were more frequently sent to nursing homes after discharge from hospital. These results can be discussed as we did above for men, as they seem to point to a greater severity and intent of the suicide attempt in this subgroup, while in those with a previous history of suicide attempt, the current one seemed less severe. Nonetheless, we know that suicide attempts represent the strongest risk factor for a later suicide death, and therefore, the suicide mortality rate increases with the number of serious attempts (41).

It is likely that different clusters and types of suicide attempters exist. In a study (albeit not specifically focused on older adults) involving suicide attempters presenting at an ER in Paris, differences were identified between multiple attempters and single attempters (42). The first seemed more likely to report previous mental healthcare (such as, mental health follow-up, psychiatric medication, or psychiatric hospitalization), but did not seem to have a stronger motive to die compared with single attempters. Another study (again, not specifically focused on older adults) identified the following clusters: attempters with poor planning and less lethal methods (“impulse-ambivalent”); attempters who have more carefully planned the attempt (“well-planned”); frequent suicide attempters, with a history of more attempts (“frequent”) (43). These studies suggests that different high-risk groups might exist (e.g., that of multiple attempters and that of individuals with a stronger desire to die), which might benefit from different treatment approaches (44).

As a significant proportion of individuals with suicidal behaviors do not seek help for their possible underlying mental health problems, help-seeking can play the role of protective factor. Nonetheless, negative attitudes and stigma still exist regarding help-seeking for mental health-related problems, hindering the likelihood of asking for help on behalf of those who need it (36). It has been previously shown that subjects with a suicidal past, compared with those without, may be less likely to seek help, both professional and informal; on the other hand, they may be more likely to experience stigma and to have more accepting attitudes toward suicide.

### Comparison Between Patients With and Without a Previous Psychiatric History Admitted to the Psychiatry Ward During the Study Period

As expected, patients with a previous psychiatric history, compared with those without, were more likely to be treated by psychiatric services and to take medications; furthermore, they were more likely to have a history of previous suicide attempts and admissions to a Psychiatry Ward.

Regarding medications, although more patients in the subgroup with a previous psychiatric history were taking psychotropic medications, up to 25% of those without a psychiatric history were taking medications as well. The common practice of prescribing anxiolytics and sedatives outside the psychiatric context and often with no proper approach to diagnosis may be particularly problematic in old age patients, considering the possible difficulties in differential diagnosis between conditions requiring or not psychiatric support and between conditions, such as dementia and depression (8, 10).

### Limitations

As already stated, the current results need to be considered with caution, in the light of the intrinsic limitations of the study design, i.e., the single-center setting and the small sample size (correlated to the specific and selected focus of our research). These limitations affect the possibility of comparisons between subgroups (also between different diagnoses), a deeper analysis of more specific socio-demographic factors (i.e., marital status and specific occupations) and the generalizability of results. Nonetheless, the Hospital where the study was performed is the second largest one in Piedmont, and has a large catchment area, which is the north-eastern part of Piedmont. Regrettably, we cannot report about the actual proportion of suicide attempters accessing the ER who are eventually admitted to our Psychiatry Ward. However, considering the interest of these preliminary results, our aim is to increase the sample size extending the assessment of charts before 2016. The possibility of performing a multi-center study would be intriguing, but feasibility need to be assessed.

Another important limitation is that we included those patients who were admitted to the Psychiatry Ward after the attempt, hence the current results are representative of a selected population of patients, which excludes those who were not referred to the Hospital and those who were not admitted to the Psychiatry Ward (e.g., those who were discharged from the ER or those who were admitted to other Hospital Wards and then discharged directly by them). Further studies exploring suicide attempt in older adults and involving the ER and other Hospital Wards could be helpful to bridge this gap.

Moreover, the small sample size did not allow to perform a multivariable analysis and consequently to assess the independent effect of patients' characteristics on gender, previous suicide attempts, and previous psychiatric history but only to generate hypothesis regarding potential relationships. Furthermore, it was not possible to determine the role played by a current or previous diagnosis of depression (considering that under this label very different types of depression may be included) and previous suicide attempts in leading to the current one.

Last, though it may not always be easy to differentiate between suicide attempt and accidental self-harm, we performed a rigorous clinical assessment to include only actual suicide attempts (4), i.e., self-injurious behaviors with least some intent to die.

## Conclusions

Suicide in older adults represents an important public health problem, considering the established fact that suicide rates grow with advancing age, (44) and, consequently, it requires a deeper investigation of modifiable risk factors in this specific population, to prevent this event, when possible.

In line with the literature, in our study we found that suicide attempters are mostly women, even in the older population, confirming the general population trend. Women appear to be more proactive in asking for help, more likely to adopt less potentially lethal methods for the suicide attempt, and more likely to be already treated by or referred to a psychiatric service. These data may suggest that more research is needed to identify a way to facilitate the access to psychiatric services for the male population aged 65 and older, or to offer support and care for the non-psychiatric reasons (comorbidities, pain, and loss of autonomy) possibly underlying suicidal behavior in older adults. In addition, our results suggest that patients who attempted suicide for the first time, compared with those with a previous history of suicide attempts, showed a stronger death proposal, choosing more lethal methods with more severe organic consequences.

The use of medications in older adults deserves more attention; suggesting that prescription may not imply a specialistic evaluation, which could be problematic considering the possible critical diagnostic issues in this age group.

Further research is needed to deepen the role of organic comorbidities, pain, disability, loss of autonomy, and the burden of other social factors, in suicidal behavior of older adults, which could not be specifically assessed in this study. From a clinical standpoint, this could offer better chances to identify potential targets for preventive interventions to address the complex topic of suicidal behaviors in this fragile and vulnerable population.

## Data Availability Statement

The raw data supporting the conclusions of this article will be made available by the authors, without undue reservation.

## Ethics Statement

Ethical review and approval was not required for the study on human participants in accordance with the local legislation and institutional requirements. Written informed consent for participation was not required for this study in accordance with the national legislation and the institutional requirements.

## Author Contributions

PZ and CG conceived, designed and coordinated the study, and drafted the manuscript. LB, MM, and MR collected data and contributed to drafting the manuscript. EG and LS performed the statistical analysis. PZ and CG coordinated data collection and revised the manuscript. All authors contributed with important intellectual content and read and approved the final manuscript.

## Conflict of Interest

The authors declare that the research was conducted in the absence of any commercial or financial relationships that could be construed as a potential conflict of interest.

## Publisher's Note

All claims expressed in this article are solely those of the authors and do not necessarily represent those of their affiliated organizations, or those of the publisher, the editors and the reviewers. Any product that may be evaluated in this article, or claim that may be made by its manufacturer, is not guaranteed or endorsed by the publisher.

## References

[1] World Health Organization. Suicide. Key facts (2021). Available online at: https://www.who.int/news-room/fact-sheets/detail/suicide. (accessed June 17, 2021)

[2] Santos J Martins S Azevedo LF Fernandes L. Pain as a risk factor for suicidal behavior in older adults: a systematic review. Arch Gerontol Geriatr. (2020) 87:104000. 10.1016/j.archger.2019.104000 31891889

[3] Fox KR Millner AJ Mukerji CE Nock MK. Examining the role of sex in self-injurious thoughts and behaviors. Clin Psychol Rev. (2018) 66:3–11. 10.1016/j.cpr.2017.09.009 28993102

[4] Carrasco-Barrios MT Huertas P Martín P Martín C Castillejos-Anguiano MC Petkari E. Determinants of suicidality in the european general population: a systematic review and meta-analysis. Int J Environ Res Public Health. (2020) 17:1–24. 10.3390/ijerph17114115 32526975 PMC7312422

[5] Okolie C Dennis M Simon Thomas E John A. A systematic review of interventions to prevent suicidal behaviors and reduce suicidal ideation in older people. Int Psychogeriatrics. (2017) 29:1801–24. 10.1017/S1041610217001430 28766474

[6] Gramaglia C Calati R Zeppegno P. Rational suicide in late life: a systematic review of the literature. Med. (2019) 55:1–23. 10.3390/medicina55100656 31569542 PMC6843265

[7] De Minayo MCS Cavalcante FG. Suicide attempts among the elderly: a review of the literature (2002/2013). Cienc e Saude Coletiva. (2015) 20:1751–62. 10.1590/1413-81232015206.10962014 26060953

[8] Wand APF Peisah C Draper B Brodaty H. Understanding self-harm in older people: a systematic review of qualitative studies. Aging Ment Heal. (2017) 22:289–98. 10.1080/13607863.2017.1304522 28326821

[9] McKee KY Kelly A. Management of grief, depression, and suicidal thoughts in serious illness. Med Clin North Am. (2020) 104:503–24. 10.1016/j.mcna.2020.01.003 32312412

[10] Draper BM. Suicidal behaviour and suicide prevention in later life. Maturitas. (2014) 79:179–83. 10.1016/j.maturitas.2014.04.003 24786686 PMC7131116

[11] Conejero I Olié E Courtet P Calati R. Suicide in older adults: current perspectives. Clin Interv Aging. (2018) 13:701–12. 10.2147/CIA.S130670 29719381 PMC5916258

[12] Goldsmith SK Pellmar TC Kleinman AM Bunney WE. National Research Council. Reducing Suicide: A National Imperative. (2002). p. 516. 25057611

[13] Chauliac N Leaune E Gardette V Poulet E Duclos A. Suicide prevention interventions for older people in nursing homes and long-term care facilities: a systematic review. J Geriatr Psychiatry Neurol. (2020) 33:307–15. 10.1177/0891988719892343 31840568

[14] Ribeiro GCA Vieira W de A Herval ÁM Rodrigues RPCB Agostini BA Flores-Mir C. Prevalence of mental disorders among elderly men: a systematic review and meta-analysis. São Paulo Med J. (2020) 138:190–200. 10.1590/1516-3180.2019.0454.r1.16012020 32491089 PMC9671226

[15] Minayo MCDS Figueiredo AEB Mangas RMDN. Study of scientific publications (2002-2017) on suicidal ideation, suicide attempts and self-neglect of elderly people hospitalized in long-term care establishments. Cienc e Saude Coletiva. (2019) 24:1393–404. 10.1590/1413-81232018244.01422019 31066841

[16] Fässberg MM Cheung G Canetto SS Erlangsen A Lapierre S Lindner R. A systematic review of physical illness, functional disability, and suicidal behaviour among older adults. Aging Ment Heal. (2016) 20:166–94. 10.1080/13607863.2015.1083945 26381843 PMC4720055

[17] Rurup ML Pasman HRW Goedhart J Deeg DJH Kerkhof AJFM Onwuteaka-Philipsen BD. Understanding why older people develop a wish to die: a qualitative interview study. Crisis. (2011) 32:204–16. 10.1027/0227-5910/a000078 21940258

[18] Balasubramaniam M. Rational suicide in elderly adults: a clinician's perspective. J Am Geriatr Soc. (2018) 66:998–1001. 10.1111/jgs.15263 29500824

[19] Conwell Y Thompson C. Suicidal behavior in elders. Psychiatr Clin North Am. (2008) 31:333–56. 10.1016/j.psc.2008.01.004 18439452 PMC2735830

[20] Zeppegno P Manzetti E Valsesia R Siliquini R Ammirata G De Donatis O. Differences in suicide behaviour in the elderly: a study in two provinces of Northern Italy. Int J Geriatr Psychiatry. (2005) 20:769–75. 10.1002/gps.1354 16035130

[21] Zeppegno P Gramaglia C di Marco S Guerriero C Consol C Loreti L. Intimate partner homicide suicide: a mini-review of the literature (2012–2018). Curr Psychiatry Rep. (2019) 21:24–9. 10.1007/s11920-019-0995-2 30788614

[22] Simon RI. Silent suicide in the elderly. Bull Am Acad Psychiatry Law. (1989) 17:83–95. 2706338

[23] Conwell Y Duberstein PR Cox C Herrmann JH Forbes NT Caine ED. Relationships of age and axis I diagnoses in victims of completed suicide: a psychological autopsy study. Am J Psychiatry. (1996) 153:1001–8. 10.1176/ajp.153.8.1001 8678167

[24] Turvey CL Conwell Y Jones MP Phillips C Simonsick E Pearson JL. Risk factors for late-life suicide : a prospective, community-based study. Am J Geriatr Psychiatry. (2002) 10:398–406. 10.1097/00019442-200207000-00006 12095899

[25] Petteri Sokero T Melartin TK Rytsälä HJ Leskelä US Lestelä-Mielonen PS Isometsä ET. Suicidal ideation and attempts among psychiatric patients with major depressive disorder. J Clin Psychiatry. (2003) 64:1094–100. 10.4088/JCP.v64n0916 14628986

[26] Richman J. The Case against Rational Suicide. Suicide Life-Threatening Behav. (1988) 18:285. 10.1111/j.1943-278X.1988.tb00165.x 3188145

[27] Cheung G Douwes G Sundram F. Late-life suicide in terminal cancer: a rational act or underdiagnosed depression? J Pain Symptom Manage. (2017) 54:835–42. 10.1016/j.jpainsymman.2017.05.004 28807701

[28] De Leo D Draper B Krysinska K Wasserman C Wasserman D. Suicidal Elderly People in Clinical and Community Settings Risk Factors, Treatment, and Suicide Prevention. Oxford Textbook of Suicidology and Suicide Prevention. (2009). p. 703–19. 10.1093/med/9780198570059.003.0095

[29] Hawton K van Heeringen K. Suicide. Lancet. (2009) 373:1372–81. 10.1016/S0140-6736(09)60372-X 19376453

[30] Zeppegno P Gramaglia C Castello LM Bert F Gualano MR Ressico F. Suicide attempts and emergency room psychiatric consultation. BMC Psychiatry. (2015) 15:1–8. 10.1186/s12888-015-0392-2 25652192 PMC4327969

[31] Mościcki EK. Epidemiology of completed and attempted suicide: toward a framework for prevention. Clin Neurosci Res. (2001) 1:310–23. 10.1016/S1566-2772(01)00032-9

[32] Cutcliffe JR Barker P. The Nurses' Global Assessment of Suicide Risk (NGASR): developing a tool for clinical practice. J Psychiatr Ment Health Nurs. (2004) 11:393–400. 10.1111/j.1365-2850.2003.00721.x 15255912

[33] SAS Institute Inc., SAS 9.1.3 Help and Documentation, Cary. NC: SAS Institute Inc., (2002-2004).

[34] Phillipe I Temesvary B Wasserman D Fricke S. Attempted suicide in Europe : rates, trend. S and sociodemogra phic characteristics of suicide attempters during the period 1989-1992. Results of the WHO/EURO Multicentre Study on Parasuicide. Acta Psychiatr Scand. (1996) 93:327–38. 10.1111/j.1600-0447.1996.tb10656.x 8792901

[35] Schrijvers DL Bollen J Sabbe BGC. The gender paradox in suicidal behavior and its impact on the suicidal process. J Affect Disord. (2012) 138:19–26. 10.1016/j.jad.2011.03.050 21529962

[36] Reynders A Kerkhof AJFM Molenberghs G Van Audenhove C. Help-seeking, stigma and attitudes of people with and without a suicidal past. A comparison between a low and a high suicide rate country. J Affect Disord. (2015) 178:5–11. 10.1016/j.jad.2015.02.013 25770477

[37] Ridge D Smith H Fixsen A Broom A Oliffe J. How men step back – and recover – from suicide attempts: a relational and gendered account. Sociol Heal Illn. (2021) 43:238–52. 10.1111/1467-9566.13216 33151571

[38] Keohane A Richardson N. Negotiating gender norms to support men in psychological distress. Am J Mens Health. (2018) 12:160–71. 10.1177/1557988317733093 29019282 PMC5734549

[39] Katz C Randall JR Leong C Sareen J Bolton JM. Psychotropic medication use before and after suicidal presentations to the emergency department: a longitudinal analysis. Gen Hosp Psychiatry. (2020) 63:68–75. 10.1016/j.genhosppsych.2018.10.003 32250247

[40] Stiawa M Müller-Stierlin A Staiger T Kilian R Becker T Gündel H. Mental health professionals view about the impact of male gender for the treatment of men with depression - a qualitative study. BMC Psychiatry. (2020) 20:1–13. 10.1186/s12888-020-02686-x 32493263 PMC7268222

[41] Liu Yanzhang Zhang Jie Sun L. Who are likely to attempt suicide again? A comparative study between the first and multiple timers. Compr Psychiatry. (2016) 176:100–106. 10.1016/j.comppsych.2017.07.007 28803042 PMC5600866

[42] Perquier F Duroy D Oudinet C Maamar A Choquet C Casalino E. Suicide attempters examined in a Parisian Emergency Department: Contrasting characteristics associated with multiple suicide attempts or with the motive to die. Psychiatry Res. (2017) 253:142–9. 10.1016/j.psychres.2017.03.035 28365537

[43] Lopez-Castroman J Nogue E Guillaume S Picot MC Courtet P. Clustering suicide attempters: impulsive-ambivalent, well-planned, or frequent. J Clin Psychiatry. (2016) 77:e711–8. 10.4088/JCP.15m09882 27035768

[44] Bertolote JM Fleischmann A. A global perspective in the epidemiology of suicide. Suicidologi. (2002). 7:3. 10.5617/suicidologi.2330

